# DOF transcription factors: Specific regulators of plant biological processes

**DOI:** 10.3389/fpls.2023.1044918

**Published:** 2023-01-20

**Authors:** Xiaoman Zou, Hongmei Sun

**Affiliations:** ^1^ Key Laboratory of Protected Horticulture of Education Ministry, College of Horticulture, Shenyang Agricultural University, Shenyang, China; ^2^ National and Local Joint Engineering Research Center of Northern Horticultural Facilities Design and Application Technology, Shenyang, China

**Keywords:** transcription factor, DOF, zinc finger, plant hormones, metabolic regulation

## Abstract

Plant biological processes, such as growth and metabolism, hormone signal transduction, and stress responses, are affected by gene transcriptional regulation. As gene expression regulators, transcription factors activate or inhibit target gene transcription by directly binding to downstream promoter elements. DOF (DNA binding with One Finger) is a classic transcription factor family exclusive to plants that is characterized by its single zinc finger structure. With breakthroughs in taxonomic studies of different species in recent years, many DOF members have been reported to play vital roles throughout the plant life cycle. They are not only involved in regulating hormone signals and various biotic or abiotic stress responses but are also reported to regulate many plant biological processes, such as dormancy, tissue differentiation, carbon and nitrogen assimilation, and carbohydrate metabolism. Nevertheless, some outstanding issues remain. This article mainly reviews the origin and evolution, protein structure, and functions of DOF members reported in studies published in many fields to clarify the direction for future research on DOF transcription factors.

## Introduction

Regulation of gene transcription is fundamental to plant germination, growth, and tissue differentiation, and numerous transcription factors (TFs) are essential for these processes (Xian et al., 2020). Transcription factors, also referred to as trans-acting factors, are DNA-binding proteins that selectively bind to cis-acting elements in the promoter regions of target genes to promote or repress their transcription (Feng et al., 2016). Therefore, the structure and function of TFs have aroused great interest from scholars and are trending topics in the field of plant molecular biology. According to their conserved domains, TFs are divided into different families, including the WRKY, NAC, bZIP, AP2/ERF, bHLH, and Cys2-His2 zinc finger (ZF) families. Different types of TFs have evolved to regulate the expression of various plant-specific genes or signals. DOF (DNA binding with One Finger) is a classic protein in the ZF superfamily (Yang et al., 2022; Zhang et al., 2022). An increasing number of members of the DOF TF family have been reported to be involved in regulating plant germination (Chen et al., 2017), growth and development (Liu et al., 2022), and responses to abiotic stress (Sun et al., 2021). A recent perspective article reported that DOFs are involved in multiple hormonal pathways during abiotic stresses (Wang et al., 2022). However, an in-depth understanding of the DOF family reveals that DOFs also play an important role in other processes in plants. To direct follow-up studies of DOF TFs, this article mainly reviews the origin and evolution, protein structure, and biological functions of DOF in various aspects of plant life.

## Origin and evolution of DOF TFs

DOF proteins were originally found in maize (Yanagisawa and Izui, 1993) but not in yeast or *Drosophila*. These proteins are members of a plant-specific TF family that has not been detected in other eukaryotes (Riechmann et al., 2000). Compared to other TFs, fewer DOF members are present in the plant genome, and significant variation has been observed across different species. Although *Chlamydomonas reinhardtii* has only one DOF transcription factor, studies have shown that DOFs seem to have originated from this copy (Moreno-Risueno et al., 2007b). Since no identifiable *DOF* gene was identified in the genomes of red algae (*Cyanidioschyzon merolae*) or diatoms (*Thalassiosira pseudonana*), researchers have proposed that the origin of DOF TFs predates the isolation of green algae and terrestrial plant ancestors (Shigyo et al., 2007). The multiplication of DOF family genes seems to be related to the emergence of various transcriptional regulatory mechanisms in plant evolution. Some DOF TFs may play conserved roles in both vascular and non-vascular plants (Sugiyama et al., 2012). Earlier studies identified 37 *DOF* members in *Arabidopsis thaliana*, one of which is a pseudogene (Yanagisawa, 2002), while 30 *DOF* members were identified in rice (*Oryza sativa*) (Lijavetzky et al., 2003). With the analysis of the whole genomes of many species, an increasing number of *DOF* members have been identified in different species (**Table 1**), including 26 *DOF* genes in birch (*Betula platyphylla*) (Sun et al., 2021), 36 in watermelon (*Citrullus lanatus*) (Zhou et al., 2020), 35 in foxtail millet (*Setaria italica*) (Zhang et al., 2017), 22 in spinach (*Spinacia oleracea*) (Yu et al., 2021a) and 24 in rose (*Rosa chinensis*) (Nan et al., 2021). Despite the species differences, the functions of DOF members in the monocot model plant rice are still questioned. In particular, the issue of functional differences or redundancy is highly controversial. Some scientists believe that the functions of DOF proteins are highly redundant (Noguero et al., 2013; Huang et al., 2020), while others argue that large differences exist (Yanagisawa, 2016; Renau-Morata et al., 2020). Huang et al. (2020) suggested that the functional redundancy of *DOF* genes in rice strain ZH11 might mask the phenotypes of individual mutants. In contrast, Yu et al. (2021b) confirm that knockout lines of all *OsDOF* family members have significant phenotypic defects in the japonica rice strain TP309, indicating that the *OsDOF* family has low redundancy. The structural diversity of DOF TFs may be closely related to complex physiological regulatory networks established during plant evolution. However, the functions of many DOFs are still unknown. Consequently, elucidating the function of each DOF protein in plants is crucial for understanding the connection between the functional diversity of plant-specific TFs and plant species evolution.

**Table 1:** Numbers of *DOF* genes in different plant species.

Non-vascular plant	No. of *DOFs*	Reference
Moss		
*Physcomitrella patens*	19	Shigyo et al., 2007
Algae		
*Chlamydomonas reinhardtii*	1	Shigyo et al., 2007
*Ostreococcus tauri*	1	Lucas-Reina et al., 2015
*Volvox carteri*	1	Lucas-Reina et al., 2015
*Micromonas pusilla*	1	Lucas-Reina et al., 2015
Vascular plants		
Fern		
*Selaginella moellendorffiii*	12	Shigyo et al., 2007
Gymnosperm		
*Pinus taeda*	10	Shigyo et al., 2007
Angiosperm		
Monocotyledons		
*Oryza sativa*	30	Lijavetzky et al., 2003
*Saccharum officinarum*	25	Gupta et al., 2014
*Hordeum vulgare*	26	Moreno-Risueno et al., 2007b
*Sorghum bicolor*	28	Kushwaha et al., 2011
*Zea mays*	18	Jiang et al., 2012
*Triticum aestivum*	96	Liu et al., 2020a
*Phyllostachys edulis*	26	Wang et al., 2016a
*Brachypodium distachyon*	27	Hernando-Amado et al., 2012
*Musa acuminata*	74	Dong et al., 2016
Dicotyledons		
*Arabidopsis thaliana*	36	Yanagisawa, 2002
*Populus trichocarpa*	41	Yang et al., 2006
*Solanum melongena*	29	Wei et al., 2018
*Jatropha curcas*	24	Wang et al., 2018
*Medicago sativa*	40	Cao et al., 2020
*Chrysanthemum morifolium*	20	Song et al., 2016
*Camellia sinensis*	16	Yu et al., 2020
*Solanum lycopersicum*	34	Cai et al., 2013
*Cucumis sativus*	36	Wen et al., 2016
*Capsicum annuum*	33	Wu et al., 2016
*Prunus persica*	25	Chen et al., 2017
*Solanum tuberosum*	35	Venkatesh and Park, 2015
*Pyrus bretschneideri*	45	Liu et al., 2020b
*Manihot esculenta*	45	Zou et al., 2019
*Brassica rapa*	76	Ma et al., 2015
*Vitis vinifera*	25	da Silva et al., 2016
*Daucus carota*	46	Huang et al., 2016
*Malus domestica*	60	Zhang et al., 2018a
*Ricinus communis*	24	Zou and Zhang, 2019
*Gossypium arboreum*	58	Chattha et al., 2020
*Gossypium hirsutum*	89	Chattha et al., 2020
*Gossypium barbadense*	110	Chattha et al., 2020

## Function of the conserved DOF domain in different plant groups

**Figure 1:**
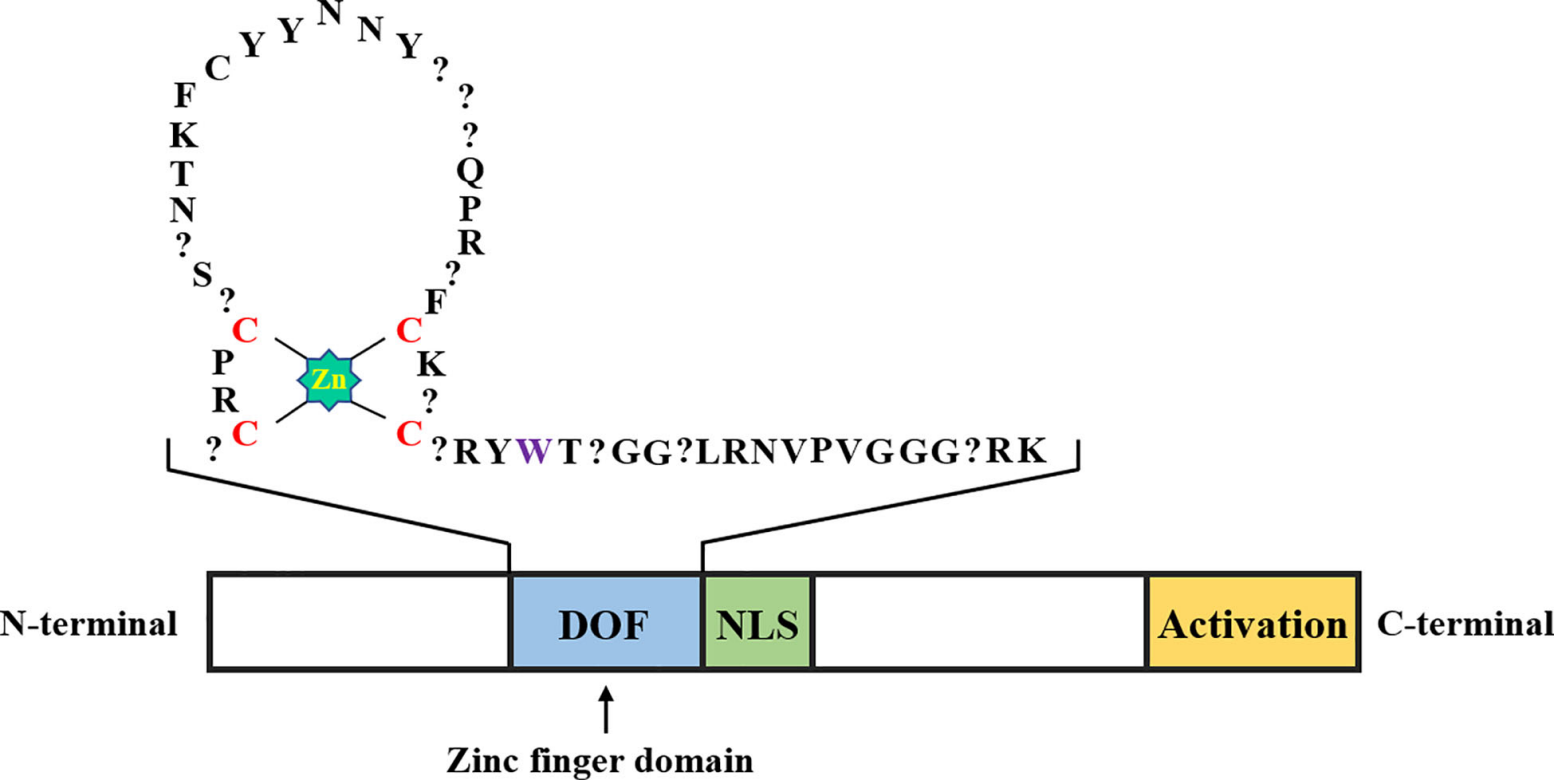
Schematic representation of the DOF transcription factor structure. The DOF zinc finger domain, nuclear localization signal, and transcriptional activation domain are shown in blue, green, and yellow, respectively. The amino acid sequence in the zinc finger domain of DOF is represented by abbreviations, the zinc coordination is shown in bright green, the position of the four cysteines in the zinc finger structure is shown in red, the question mark represents any amino acid, and the tryptophan residue mentioned in the second part is shown in purple.

DOF proteins consist of 200-400 amino acids, and a conserved DOF domain with a zinc finger (ZF) structure containing 50-52 amino acid residues is located at its N-terminal region, along with a transcriptional regulatory domain in the C-terminal region (**Figure 1**). Unlike other ZF proteins, DOF TFs contain only one Cys2/Cys2 ZF, and the ZF structure specifically recognizes the upstream core sequence 5’-(T/A)/AAAG-3’ of the target gene (Umemura et al., 2004; Kim et al., 2010). More specifically, the pumpkin DOF protein AOBP recognizes the AGTA motif and plays a role in facilitating binding of this protein to DNA (Kisu et al., 1998). The positions of four cysteines that comprise the ZF structure are necessary to achieve loop stability. The tryptophan in the C-terminal region of the ZF protein is important for DNA binding. In steroid hormone receptors, the tryptophan residue seems to play a role in stabilizing the structure (Noguero et al., 2013). However, the affinity of the conserved ZF domain in the DOF protein for AAAG motifs of downstream target genes is unknown. Moghaddas Sani et al. (2018) determined the binding affinity between the DOF-ZF domain and target oligonucleotides in *Arabidopsis thaliana* using microscale thermophoresis. The authors proposed that when two binding sites are present, the affinity of the DOF domain for an oligonucleotide is 100 times higher than that for the single binding site. This result explains why many repeated AAAG motifs exist in the promoters of downstream target genes of DOF TFs. Compared to other TF families, DOF proteins recognize relatively shorter motifs (Umemura et al., 2004), which has led to the discovery of many putative DOF binding sites in the promoter regions of many genes. However, most sites may be non-functional *in vivo*, and accumulating evidence suggests that the location of the AAAG motif limits DOF protein binding to DNA (Cavalar et al., 2003). Thus, DOF proteins may need to interact with other TFs to ensure the precise targeting and binding of DNA and subsequently promote transcription.

The presence of two distinct domains in the N-terminal and C-terminal regions indicates that the DOF proteins are bifunctional (Noguero et al., 2013; Zou et al., 2019). The C-terminal region of DOF proteins contains a nonconserved domain that may interact with various TFs, including WRKY, bZIP, and MYB proteins, and thus function at different levels of expression (Zhang et al., 1995; Isabel-LaMoneda et al., 2003; Zou et al., 2008). The *Arabidopsis* DOF protein OBP1 was shown for the first time to specifically enhance the binding of the bZIP transcription factor OBF to its DNA target OSC element through a protein-protein interaction (Zhang et al., 1995). Subsequently, the ZmDOF2 protein in maize was shown to interact with HMG (High Mobility Group) proteins and affect the binding efficiency to downstream target sites (Yanagisawa, 1997). The barley DOF protein BPBF has been reported to interact with the MYB transcription factor GAMYB to activate the promoter of the target gene (Diaz et al., 2002). In addition, a binary nuclear localization signal (NLS) is located between the two domains of DOF proteins, which may partially overlap with them (Krebs et al., 2010).

## Functions of DOFs in plant dormancy and germination

Dormancy and germination are complex multistage processes in the plant life cycle that are tightly regulated by the coordinated expression of various genes in different tissues. As key TFs regulating gene expression, studies of DOF proteins are crucial for analyzing the molecular mechanism and regulatory network of plant dormancy and germination. In monocotyledons, the barley DOF proteins HvDOF17 and HvDOF19 were shown to regulate the expression of aleurone hydrolase genes and induce seed germination (Diaz et al., 2005; Moreno-Risueno et al., 2007a). An earlier study reported that OsDOF3 may be a mediator of GA signaling during rice germination (Washio, 2001). Some DOF TFs have been reported to regulate seed germination in response to exogenous GA_3_ in barley (Mena et al., 2002) and rice (Washio, 2003), but the regulatory mechanism between DOF and GA signaling remains unknown. The DOF protein DAG1, which affects germination, was detected in the dicotyledonous model plant *A. thaliana* and shown to play a key role in the establishment and maintenance of seed dormancy by controlling the dynamic balance of GA and ABA (Gabriele et al., 2010). However, the molecular mechanism by which DOF regulates seed germination has not been fully resolved. Subsequently, DAG1 was reported to interact with the DELLA protein GAI to inhibit the expression of the GA biosynthesis gene *AtGA3ox1* and maintain seed dormancy (Boccaccini et al., 2014). The stability of the DAG1 protein was also controlled by GA (Boccaccini et al., 2016). Unlike DAG1, *Arabidopsis* DAG2 plays an active role in seed germination but is negatively regulated by DAG1 (Santopolo et al., 2015). As more DOF members were identified, AtDOF6 was shown to interact with AtTCP14 and inhibit seed germination (Rueda-Romero et al., 2012). Likewise, AtDOF6 interacted with the DELLA protein RGL2 to form the RGL2-DOF6 complex, which bound to the downstream *AtGATA12* promoter and promoted seed dormancy (Ravindran et al., 2017). In addition to seed dormancy, DOF has also been reported to regulate bud dormancy in woody plants. For example, through transcriptome analysis of European beech (*Fagus sylvatica*) (Lesur et al., 2015) and whole-genome analysis of peach (Chen et al., 2017), researchers found that the DOF family may be involved in regulating plant bud dormancy. However, the molecular mechanism by which DOF members regulate bud dormancy and the potential ability of DOF proteins to regulate geophyte dormancy still remain to be determined.

## Functions of DOFs in plant growth and development

DOF members are involved in regulating the growth and developmental stages of many plant tissues, such as root growth, hypocotyl elongation, plant morphogenesis, leaf development, and floral organ development. The tobacco (*Nicotiana tabacum*) DOF protein NtBBF1 binds to the *rolB* promoter in an auxin-induced manner, thus regulating root growth (Baumann et al., 1999). *Arabidopsis cycling DOF factor 4* (*AtCDF4*) can promote the differentiation of root columella stem cells, but this effect is inhibited by WOX5 (WUSCHEL HOMEOBOX 5) *via* recruitment of TPL/TPR co-inhibitors and histone deacetylase HDA19 (Pi et al., 2015). Between the cotyledon and the root, the part near the root is called the hypocotyl. The *Arabidopsis* DOF ​​TF COG1 (COGWHEEL1) regulates BR biosynthesis and ultimately promotes hypocotyl growth by binding to the *PIF4* and *PIF5* promoters and inducing their expression (Wei et al., 2017). *Arabidopsis* DAG1 can directly bind to the promoters of the downstream ethylene-related gene *ERF2* (*ETHYLENE RESPONSE FACTOR2*), auxin-responsive gene *SAUR67* (*SMALL AUXIN UP RNA 67*), and *WRKY18* gene, which are involved in ABA signaling to promote hypocotyl elongation (Lorrai et al., 2018). *Arabidopsis AtCDF1* and *AtCDF5* promote hypocotyl elongation under short-day conditions (Martín et al., 2020). A recent study showed that *Arabidopsis* CDF2 and PIF4 (PHYTOCHROME-INTERACTING FACTOR 4) form a complex that co-regulates the downstream target gene *YUCCA8*, thereby promoting hypocotyl elongation (Gao et al., 2022). Plants activate phytochromes or phototropin and cryptochrome light receptors *in vivo* following exposure to light to promote growth and development. *StCDF1* was active during potato tuber development under short-day (SD) conditions (Kondhare et al., 2019). *Arabidopsis* AtCOG1 depends on phytochromes upon light induction but plays a negative regulatory role in the phytochrome signaling pathway (Park et al., 2003). Interestingly, the antagonistic effect between phytochromes and COG1 may be explained in terms of the inhibition and activation of gibberellin, respectively. Phytochromes inhibit GA biosynthesis (Hedden and Thomas, 2012), while *COG1* overexpression increases endogenous GA_1_ levels in *Arabidopsis* siliques by promoting the expression of the gibberellin biosynthetic gene *GA3ox3*, which is ultimately involved in seed coat development (Bueso et al., 2016).

Studies examining plant structural regulation have shown that overexpression of *AtDOF5.4/OBP4* resulted in dwarfing of *Arabidopsis* plants by reducing cell size and number (Xu et al., 2016). Similarly, overexpression of *OsDOF12* may lead to structural changes in rice, such as a reduced plant height, erect leaves, smaller leaves, and shortened panicles, by inhibiting BR signaling (Wu et al., 2015). As shown in the study by Nilsen et al. (2020), wheat *TdDOF* may regulate stem structure by directly or indirectly downregulating the *NAC* and *CEP* genes involved in programmed cell death (PCD) and may also affect responses to oxidative stress, metal ion transport, cell wall modification, and cation transport.

According to a previous study, maize DOF1 was involved in regulating leaf development in a light-dependent manner (Yanagisawa and Sheen, 1998). As an important component of the plant foliar epidermis, guard cells control the opening or closing of stomata. The DOF transcription factor SCAP1 (STOMATAL CARPENTER 1) was shown to play a major role in the critical period of stomatal guard cell differentiation and directly contribute to the maturation of guard cells in *Arabidopsis* (Negi et al., 2013). Subsequent results indicated that *SCAP1* overexpression increased the stomatal density and index in *Arabidopsis* (Castorina et al., 2016). The functions of DOF members in the development of leaf veins have also attracted much attention. *Arabidopsis* VDOF1 (VASCULAR-RELATED DOF1) and VDOF2 (VASCULAR-RELATED DOF2) may inhibit cotyledon vein formation and lignin deposition by regulating brassinosteroid (BR) signaling and lignin-related gene transcription in inflorescence stems (Ramachandran et al., 2020). Overexpression of *AtDOF5.8* has been found to inhibit the formation of higher-order veins in cotyledons and leaves (Konishi and Yanagisawa, 2015). Using single-cell sequencing, Liu et al. (2022) identified cell types in 10 cell clusters. They elucidated the potential functions of AtCDF5 and the DELLA protein RGA in early cotyledon vein development through the TF network. Furthermore, by obtaining the *cdf5* mutant and analyzing the DAP-seq results, the authors inferred that CDF5 might control the expression of the downstream target genes *BZIP9*, *SWEET11*, *SWEET12*, and *SULTR2;1* to regulate leaf vein function. Overexpression of *AtCDF4* has been reported to impair the vein development of 3-day-old cotyledons, indicating that CDF4 is also involved in regulating cotyledon vein development (Liu et al., 2022). Additionally, DOFs have also been shown to participate in JA-induced leaf senescence in monocotyledons and dicotyledons. For example, in rice, OsDOF24 was found to delay leaf senescence by suppressing the activity of the *OsAOS* gene related to JA biosynthesis (Shim et al., 2019). However, *Arabidopsis* AtDOF2.1 has been reported to play an active role in JA-induced leaf senescence through the MYC2-DOF2.1-MYC2 feed-forward transcription loop (Zhuo et al., 2020).

Auxin can control the expression of *AtDOF2.1*, *AtDOF4.6*, *AtDOF5.3*, and *AtDOF5.8* in *Arabidopsis*, which are crucial for vascular development (Gardiner et al., 2010; Konishi et al., 2015). Interestingly, both auxin and other hormones regulate *DOFs.* The expression of the *AtDOF2.4/PEAR1* (*PHLOEM EARLY DOF1*), *AtDOF5.1/PEAR2* (*PHLOEM EARLY DOF2*), *AtDOF3.2/DOF6*, *AtDOF5.3/TMO6*, *AtDOF1.1/OBP2* (*OBF BINDING PROTEIN2*) and *AtDOF5.6/HCA2* (*HIGH CAMBIAL ACTIVITY2*) genes was reported to be regulated by auxin and cytokinin in the sieve tube of the primary phloem (Miyashima et al., 2019; Smetana et al., 2019). Downregulation of tomato *SlDOF10* triggered parthenocarpic development while suppressing vascular tissue development (Rojas-Gracia et al., 2019). *MdDOF15*, *MdDOF18*, and *MdDOF48* were significantly upregulated in apple during pollen tube growth and development (Yang et al., 2018b). Moreover, DOF members also play vital roles in regulating floral organ development. *LOW* (*LOVE ON WINGS*) is a legume *DOF* gene that controls floral organ differentiation by regulating the floral vasculature pattern and petal asymmetry of mung beans (Guo et al., 2019).

## Functions of DOFs in regulating plant flowering and fruit ripening

Floral transition is one of the most important biological processes, ensuring reproductive success by integrating internal and external signals in plants. Overexpression of the oilseed rape *BnCDF1* gene in *Arabidopsis* reduced the expression of the signal integration factors CO (CONSTANS) and FT (FLOWERING LOCUS T) in the photoperiod flowering pathway, thus delaying the flowering time (Xu and Dai, 2016). Similarly, overexpression of pear *PbDOF9.2* in *Arabidopsis* also resulted in a delayed flowering time. PbDOF9.2 increased the activity of the *PbTFL1a* and *PbTFL1b* promoters by inhibiting FT expression and inhibiting flowering (Liu et al., 2020b). Overexpression of *Medicago MtCDFd1-1* (Zhang et al., 2019) and tomato *SlCDF3* (Xu et al., 2021) also resulted in delayed flowering. In contrast, overexpression of moso bamboo *PheDOF12-1* caused *Arabidopsis* to flower earlier under long-day (LD) conditions. PheDOF12-1 regulated the flowering time by combining with the downstream *PheCOL4* promoter (Liu et al., 2019). Similarly, *OsDOF4* also promoted rice flowering under LD conditions (Wu et al., 2017a). In contrast, studies in pea (*Pisum sativum*) revealed that a *CDF* homolog *LATE2* (*LATE BLOOMER2*), which functions downstream of light signaling and the biological clock, repressed the expression of *FTb2*, an FT gene regulated by the pea photoperiod, ultimately caused a late-flowering phenotype (Ridge et al., 2016). *TaDOF5*, *TaDOF16*, and *TaDOF19* were found to be homologous to *Arabidopsis CDF* in wheat, and diurnal changes in their expression during the light-dark cycle were similar to the pattern of *CDF*, presumably indicating that these genes have potential roles in the photoperiodic regulation of flowering (Shaw et al., 2009). AtDOF4.7 caused flower organ abscission defects by inhibiting the expression of cell wall hydrolase genes (Wei et al., 2010). Furthermore, *AtDOF4.7* might be affected by ethylene and short peptide IDA (INFLORESCENCE DEFICIENT IN ABSCISSION) to negatively regulate floral organ abscission (Wang et al., 2016b). Conversely, AtCDF4 was reported to contribute to floral organ abscission in *Arabidopsis* (Xu et al., 2020).

Exogenous GA_3_-induced expression of *DOFs* has been proven to be a key factor in the initiation of tomato fruit formation (Rojas-Gracia et al., 2019). Furthermore, overexpression of *SlCDF4* induced high expression of *GA20ox* and *GA3ox* and increased tomato fruit size by regulating endogenous GA_4_ biosynthesis (Renau-Morata et al., 2020). The high expression of *DzDOF2.2* in durian increased the level of auxin and upregulated ethylene biosynthesis through the transcriptional activation of the ACC synthase gene, thus initiating auxin-ethylene crosstalk in advance and ultimately promoting early fruit ripening (Khaksar et al., 2019). Similarly, banana MaDOF23 interacted with MaERF9 to regulate fruit ripening (Feng et al., 2016). Although ethylene induced *MaDOF23* expression, MaDOF23 in turn inhibited the expression of maturity-related genes. Based on these findings, MaDOF23 acted as a repressor to fine-tune ethylene biosynthesis during fruit ripening, possibly by balancing the induction of transcriptional activators such as MaERF9. Another study showed that the downregulation of *StCDF1* affected potato propagation and the internal water balance by activating downstream *StFLORE* expression (Ramírez et al., 2021). In addition, overexpression of *AtDOF4.2* or its homologous gene *AtDOF4.4* increased the seed number and silique size (Zou et al., 2013). DASH (DOF Acting in Seed embryogenesis and Hormone accumulation) is a DOF member unique to the endosperm. Auxin transport in the *dash* mutant is impaired, resulting in embryonic defects and affecting seed size in *Medicago truncatula* (Noguero et al., 2015). It can be seen that some DOFs can also affect fruit size, quantity, and quality to a certain extent, and DOF functions must be clarified to improve crop yields and promote the development of the fruit and vegetable industry.

## Responses of DOFs to biotic and abiotic stresses

**Figure 2:**
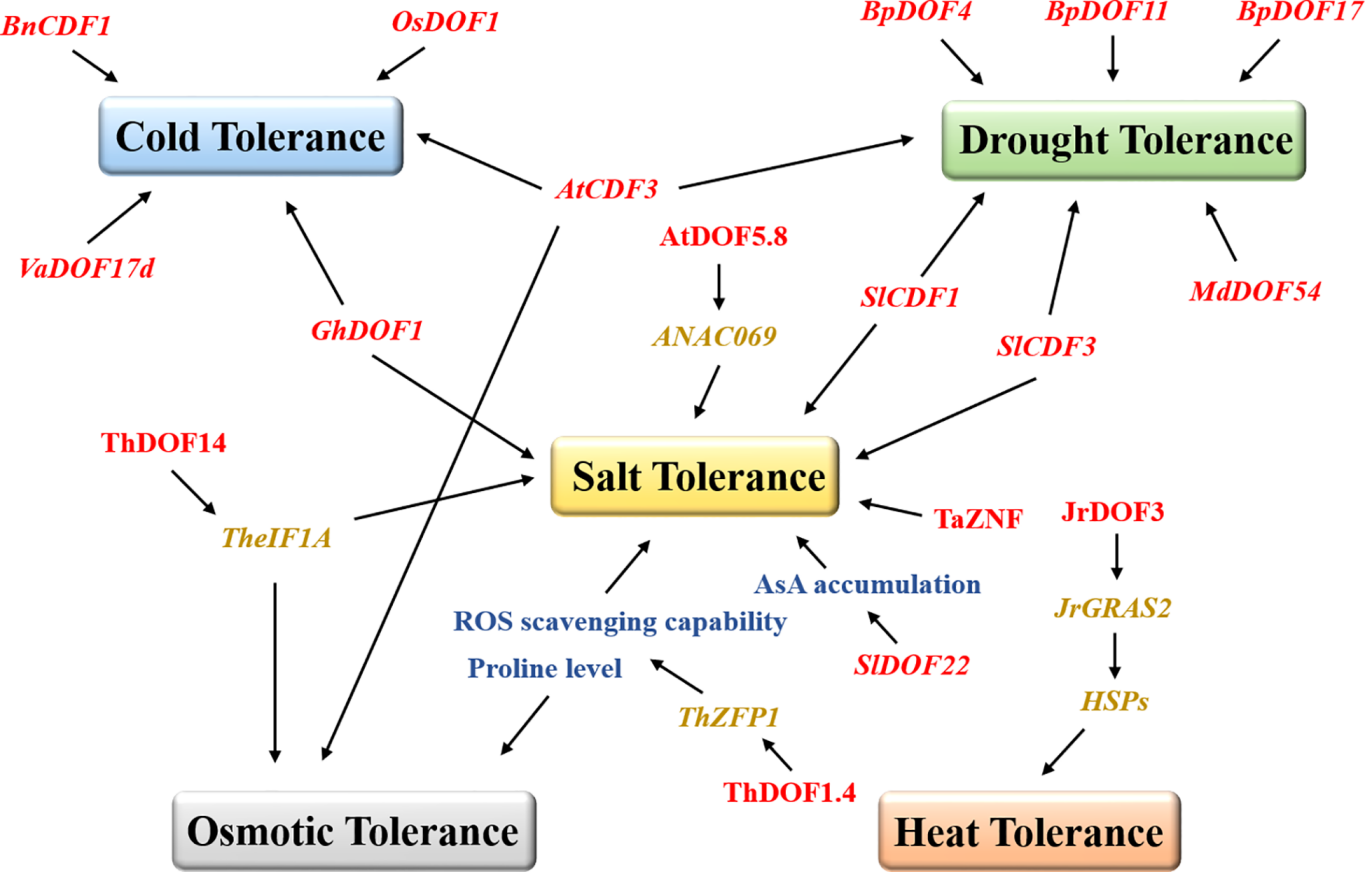
DOFs promote plant tolerance under different abiotic stresses. The DOF family members are represented in red, and the box marks resistance to different stresses. The blue, green, yellow, grey, and orange boxes represent cold tolerance, drought tolerance, salt tolerance, osmotic tolerance, and heat tolerance, respectively. The black arrows indicate facilitated relationships. AsA, ascorbic acid.

Several results suggest that DOF TFs respond to biotic stresses by improving the ability of plants to defend against pathogens. Transient expression of the *DOF* genes *BBF2* and *BBF3* in tobacco increased plant resistance to pathogens (Sasaki et al., 2015). Overexpression of grape *VvDOF3* can improve plant resistance to the powdery mildew pathogen *Golovinomyces cichoracearum* (Yu et al., 2019). However, more studies have suggested that DOF proteins resist various abiotic stresses in plants (**Figure 2**). ThDOF1.4 improved tolerance to salt and osmotic stress by increasing the proline level and improving the ROS scavenging capability of *Tamarix hispida* (Zang et al., 2017). Likewise, ThDOF14 can bind specifically to the DOF motif in the downstream *TheIF1A* promoter and may participate in plant salt stress and osmotic stress responses by regulating or interacting with TheIF1A (Yang et al., 2017). Tomato *SlDOF22* affected ascorbic acid (AsA) accumulation and improved salt tolerance in plants (Cai et al., 2016). The wheat DOF protein TaZNF increased Na^+^ excretion by controlling the expression of many downstream genes and ultimately significantly improved salt tolerance (Ma et al., 2016). *Arabidopsis* AtDOF5.8 regulated the expression of *ANAC069* and played a role in the salt signaling pathway (He et al., 2015). Similarly, watermelon *ClDOF29* was the main regulator of the salt stress response (Zhou et al., 2020). In banana, many *MaDOF* genes were affected by salt and drought stresses, resulting in downregulated expression levels (Dong et al., 2016). In contrast, the alfalfa *MsDOF10*, *MsDOF35*, and *MsDOF39* genes were significantly upregulated under the same drought and salt stress conditions (Cao et al., 2020). As a DOF transcription factor, CDF has been reported to be widely involved in the responses to various abiotic stresses in plants. The mutant *cdf3-1* gene rendered *Arabidopsis* sensitive to drought and cold stresses, while overexpression of this gene unexpectedly increased plant resistance to osmotic stress (Corrales et al., 2017). Likewise, overexpression of tomato *SlCDF1* or *SlCDF3* also improved the salt tolerance and drought resistance of plants (Corrales et al., 2014). Moreover, DOF family genes also play prominent roles in improving the drought resistance of woody plants. Overexpression of apple *MdDOF54* resulted in a higher photosynthetic rate and stronger branch water transport capacity under long-term drought conditions than the wild-type lines but substantially increased the survival rate under short-term drought conditions (Chen et al., 2020). Undoubtedly, *MdDOF54* could improve the drought tolerance of plants. Similarly, overexpression of the birch *BpDOF4*, *BpDOF11*, and *BpDOF17* genes improved drought tolerance by enhancing the ROS scavenging ability (Sun et al., 2021). Notably, DOF proteins can also improve plant survivability in extreme temperature environments. In *Brassica*, *BnCDF1* expression was induced at low temperatures, while overexpression of *BnCDF1* improved plant cold tolerance (Xu and Dai, 2016). Similarly, overexpression of cotton *GhDOF1* (Su et al., 2017), grape *VaDOF17d* (Wang et al., 2021), and rice *OsDOF1* (Liu et al., 2021) significantly enhanced plant cold tolerance. One study has shown that walnut JrDOF3 directly regulates the transcription of its target gene *JrGRAS2*, effectively regulates the expression of heat shock protein genes (HSPs), and ultimately improves heat tolerance (Yang et al., 2018a). In Chinese cabbage (Ma et al., 2015), chrysanthemum (Song et al., 2016), and spinach (Yu et al., 2021a), the expression of *DOFs* has also been found to be regulated by salt stress, cold stress, and heat stress.

**Figure 3:**
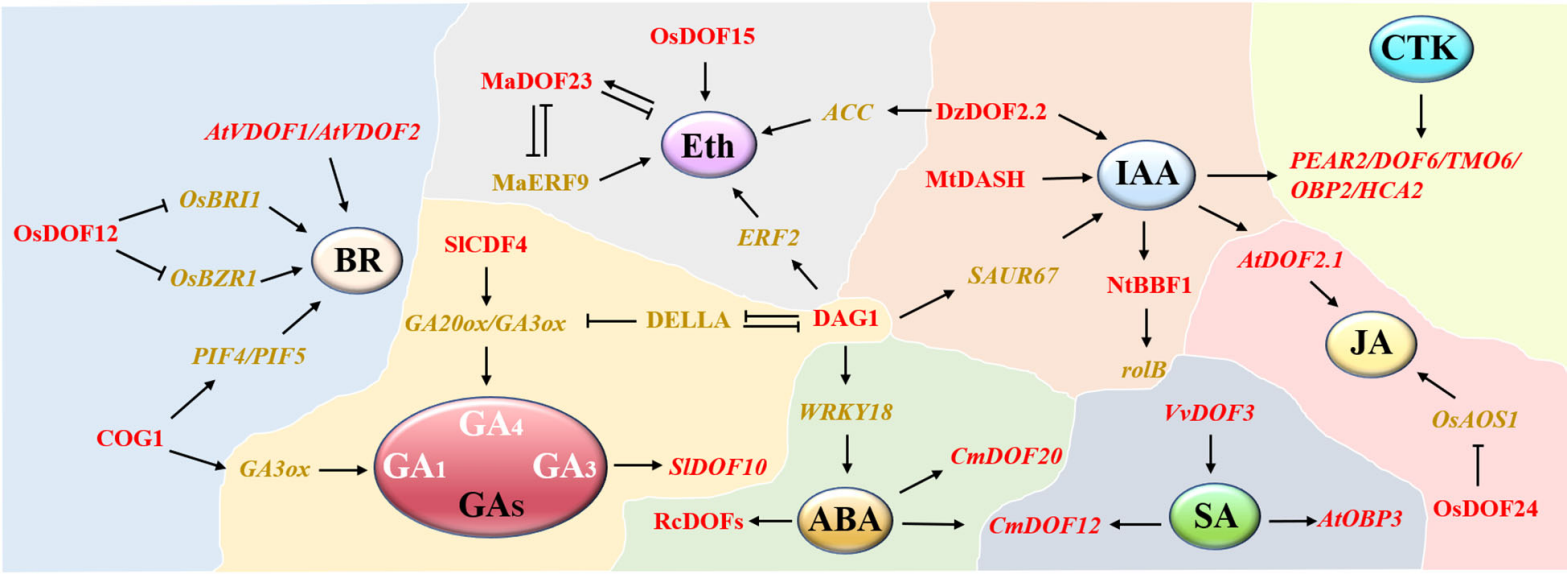
A network model of DOF members participating in different phytohormone pathways. A total of 8 hormone modules are distributed. DOF members are shown in red. Positive and negative regulatory relationships are represented by black arrows and blunted lines, respectively.

In addition, some DOF proteins are widely involved in plant stress responses by responding to some plant hormone signals, such as ABA and SA (**Figure 3**). Many *RcDOFs* in castor were expressed at two different levels in response to ABA treatment (Jin et al., 2014). Similarly, researchers have also proposed that chrysanthemum *CmDOFs* may be involved in the response to ABA and SA, thus resulting in different expression patterns, in which *CmDOF12* and *CmDOF20* are significantly upregulated by exogenous ABA, while the expression levels of *CmDOF2, CmDOF5*, *CmDOF6*, *CmDOF10*, and *CmDOF12* are upregulated by SA (Song et al., 2016). *CmDOF12* is a homolog of *Arabidopsis OBP3* (*AtDOF3.6*), consistent with the previous result that *Arabidopsis OBP3* expression was upregulated by SA induction (Kang and Singh, 2000). DOF proteins may improve the submergence tolerance of rice at the early stage of germination by regulating GA and other plant hormones (Mohanty, 2021). OsDOF15 contributes to the inhibition of rice primary root elongation under salt stress by mediating ethylene biosynthesis (Qin et al., 2019). Clearly, DOF family genes play vital roles in plant abiotic stresses, and many members are involved in regulating multiple stress pathways simultaneously. An in-depth exploration of the DOF regulatory network will help to reveal the mechanism of the plant abiotic stress response.

## Functions of DOFs in regulating plant metabolism

**Figure 4:**
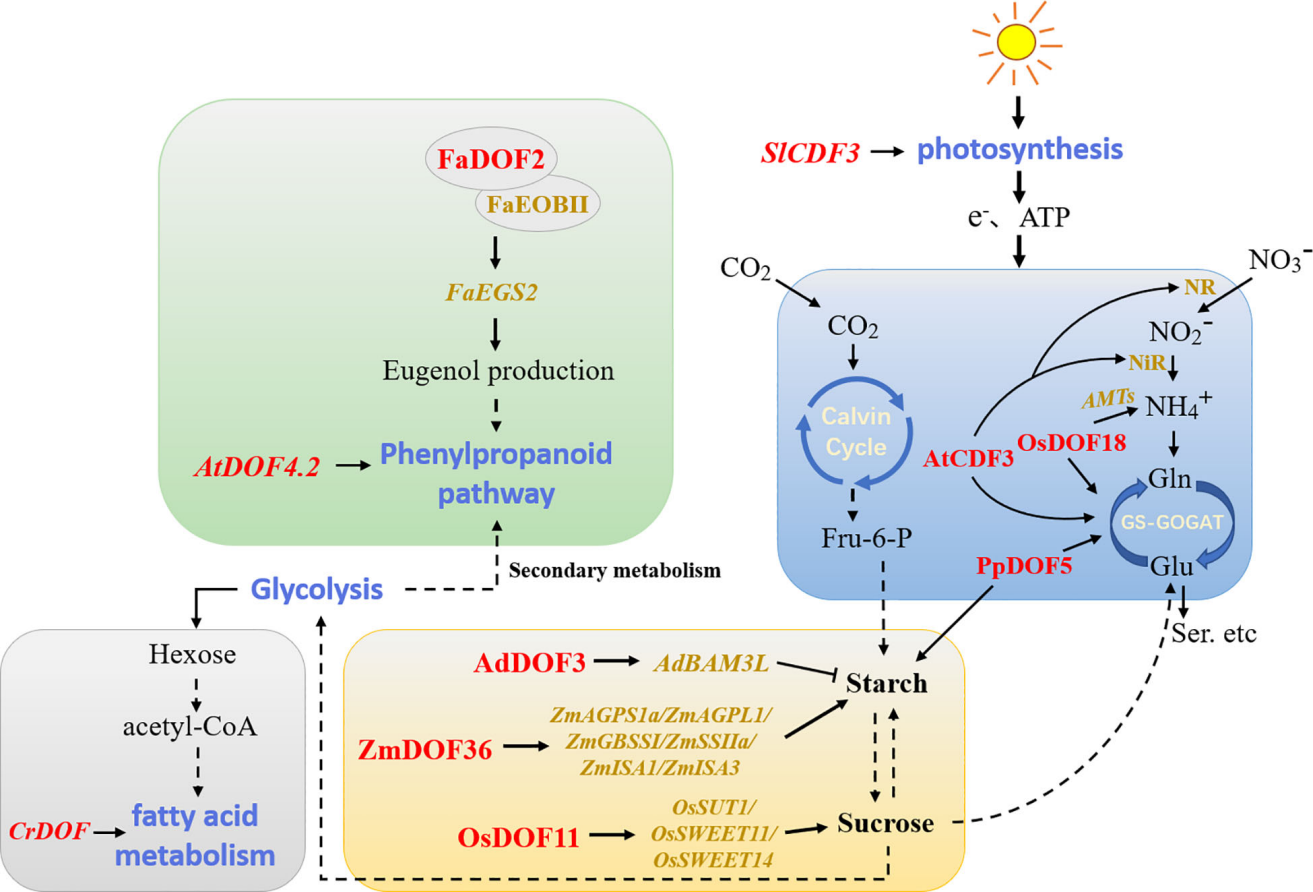
Model of DOFs involved in the regulation of plant metabolism. The blue, yellow, grey, and green modules represent carbon and nitrogen assimilation, carbohydrate metabolism, fatty acid metabolism, and phenylpropanoid metabolism, respectively. Red indicates DOF members in different plants. Dotted lines indicate that some processes have been omitted. NR/NiR, nitrate/nitrite reductases; AMTs, ammonium transporters; GS, glutamine synthetase; GOGAT, glutamate synthase; Fru-6-P, fructose-6-phosphate.

Plant metabolic regulation includes primary metabolic processes that supply the immediate needs for plant growth and secondary metabolic processes that regulate the interaction between plants and the environment. DOF members have been reported to be involved in plant metabolic regulatory networks (**Figure 4**). The metabolism of non-structural carbohydrates in plants affects growth, development, and responses to environmental factors to a great extent. Overexpression of the *DOF* gene *SRF1* in sweet potato promoted the formation of dry matter in storage roots, led to the decreases in the glucose and fructose contents, and ultimately regulated carbohydrate metabolism by inhibiting the expression of the vacuolar invertase gene (Tanaka et al., 2009). Likewise, overexpression of *PpDOF5* in transgenic hybrid poplars significantly increased carbohydrate accumulation (Rueda-López et al., 2017). A study of maize found that *ZmDOF3* knockdown inhibited the expression of genes involved in starch synthesis (Qi et al., 2017). Similarly, overexpression of *ZmDOF36* decreased the contents of reduced sugars and soluble sugars in maize seed endosperm, and the presence of higher soluble sugar levels led to starch synthesis. ZmDOF36 positively regulated the expression of different starch synthesis-related genes, such as *ZmAGPS1a*, *ZmAGPL1*, *ZmISA1*, *ZmISA3*, *ZmGBSSI*, and *ZmSSIIa*, by binding to specific motifs in downstream promoters (Wu et al., 2019). In contrast, kiwifruit (*Actinidia deliciosa*) AdDOF3 affected fruit flavor by transactivating the starch degradation gene *AdBAM3 L* upon binding to downstream AAAG/CTTT elements (Zhang et al., 2018b). Furthermore, studies in *Arabidopsis* showed that AtDOF4.6/VDOF1 and AtDOF1.8/VDOF2 modulate secondary cell wall (SCW) properties related to glycation efficiency (Ohtani et al., 2017). Rice OsDOF11 promoted sucrose transport by directly regulating the transcription of the downstream sucrose transport-related genes *OsSUT1*, *OsSWEET11*, and *OsSWEET14* (Wu et al., 2018). In addition, the only DOF protein present in *C. reinhardtii* was involved in regulating fatty acid metabolism (Ibáñez-Salazar et al., 2014). The results reported by Jia et al. (2019) confirmed that overexpression of *Chlamydomonas CrDOF* could regulate the expression of crucial genes related to lipid metabolism and promote intracellular lipid accumulation. Overexpression of soybean *GmDOF4* and *GmDOF11* increased the seed lipid content (Wang et al., 2007). Similarly, overexpression of *GmDOF4* in *Chlorella ellipsoidea* increased the cellular lipid content by approximately 50% (Zhang et al., 2014). These results provide new methods for the future development of the food processing and biofuel industries.

The antagonistic relationship between carbon and nitrogen also regulates various developmental processes in plants. A recent study has shown that OsDOF11 regulates nitrogen metabolism by affecting sugar transport and distribution, thereby coordinating the balance of carbon and nitrogen to maintain plant life processes (Huang et al., 2021). OsDOF18 was reported to regulate ammonium transport by inducing the expression of ammonium transporter genes, thus affecting the efficiency of nitrogen assimilation (Wu et al., 2017b). Maritime pine (*Pinus pinaster*) PpDOF5 was also crucial in controlling conifer ammonium assimilation for glutamine production (Rueda-López et al., 2008). *Arabidopsis* AtCDF3 promoted root development by regulating the expression of genes related to carbon and nitrogen assimilation (Domínguez-Figueroa et al., 2020). To some extent, improving the nitrogen use efficiency or photosynthetic rate of plants is the key factor in increasing crop yields. Overexpression of *SlCDF3* in tomato increased the photosynthetic rate, which also resulted in higher sucrose utilization and changes in plant primary metabolism while enhancing nitrogen assimilation, promoting an increase in biomass, and ultimately increasing yields (Renau-Morata et al., 2017). Moreover, some DOF members are also involved in secondary plant metabolism. *Arabidopsis AtDOF4.2* regulated phenylpropane metabolism in different tissues and under various stresses (Skirycz et al., 2007). FaDOF2 regulated the volatile phenylpropanoid pathway in ripe strawberry (*Fragaria* × *ananassa*) receptacles by controlling the expression of the *FaEGS2* and *FaEOBII* genes, which are involved in eugenol synthesis (Molina-Hidalgo et al., 2017). This regulation of volatile organic compounds may become a breakthrough in analyzing the mechanism of plant aroma formation.

## Conclusions and future prospects

The large number of DOF functional studies summarized here indicates that many unanswered questions about DOF TFs still remain. Although the functional diversity of DOF has attracted increasing attention in recent years, research on the molecular mechanisms by which these proteins regulate certain biological processes, such as plant dormancy and germination, has been limited to some model plants and thus further studies are needed. Some DOFs coordinate internal factors such as phytochromes and plant hormones to affect plant growth and development upon light induction, which also provides an important reference for the analysis of light signaling pathways in non-model plants. Accumulating evidence suggests that DOFs respond to a variety of plant hormone signals and participate in regulating plant growth and development, stress response, metabolic senescence, and other life stages. However, further studies are needed to explore the roles of DOFs in some phytohormone signaling pathways and determine which members are central to integrating the crosstalk between different hormones. Based on species specificity, substantial differences in bioactive GA types regulated by various DOFs exist, which provides a new direction for traditional gibberellin pathway research. The establishment of a more comprehensive DOF-GA regulatory network is an urgent need.

To date, despite our keen interest in *DOF* genes, only half of the DOF members have been characterized in model plants, and thus, our knowledge of DOF transcription factors is limited. Although some variation in the number of DOFs has been observed in different species, the potential functional redundancy in these DOF family genes still must be further confirmed by obtaining multiple gene knockout lines using CRISPR and other techniques. Some DOF members may become potential factors to improve crop yields in the future and have quite wide application prospects for the development of the food processing and biofuel industries. Accordingly, in-depth research on DOF TFs must be conducted in many fields to reveal their possible novel functions that will help us understand the intricate life processes of plants.

## Author contributions

XZ and HS conceived the structure and content of the article, and finished the manuscript. All authors contributed to the article and approved the submitted version.
